# *In-Vitro* Assessment of Magnetic Dextran-Spermine Nanoparticles for Capecitabine Delivery to Cancerous Cells

**Published:** 2017

**Authors:** Maryam Ghadiri, Ebrahim Vasheghani-Farahani, Fatemeh Atyabi, Farzad Kobarfard, Hossein Hosseinkhani

**Affiliations:** a *Biomedical Engineering Division, Faculty of Chemical Engineering, Tarbiat Modares University, Tehran, Iran. *; b *Nanotechnology Research Centre, Faculty of Pharmacy, Tehran University of Medical Sciences, Tehran, Iran.*; c *Department of Medicinal Chemistry, School of Pharmacy, Shahid Beheshti University of Medical Sciences, Tehran, Iran. *; d *Innovation Center for Advanced Technology, Matrix, Inc., New York 10029, USA.*

**Keywords:** Cancer, Controlled release, Drug delivery systems, Factorial design, U87MG

## Abstract

Cationic polymeric nanoparticles have great potential for developing drug delivery systems with limited side effects for tumor medication. The goal of this research is investigation of cationic dextran-spermine polymer (DS) efficacy for improvement of hydrophilic drug delivery to negatively charged cancerous cells. Capecitabine (as a hydrophilic antineoplastic drug) was loaded into the magnetic dextran-spermine nanoparticles (DS-NPs) via ionic gelation. Design of experiments was applied to specify how the significant factors affect size, surface charge and capecitabine entrapment efficiency of the DS-NPs. Physicochemical properties, *in-vitro* release profile and cellular studies of the optimized DS-NPs were evaluated. The experimental results indicated that DS-NPs with favorable properties can be achieved at an optimized condition of 2 mg/mL DS and 0.75 mg/mL tri-polyphosphate (TPP) concentrations, TPP addition rate of 35 mL/min, pH 3 of DS solution and super paramagnetic iron oxide nanoparticles (SPION)/DS mass ratio of 0.5. The entrapment efficiency of capecitabine was 26.1% at optimum condition and drug release at neutral pH after 24 h and acidic pH within 3 h was 56 and 98%, respectively. The cytotoxicity assessment exhibited that capecitabine loaded DS-NPs was more toxic than corresponding free drug as control. Significant cellular uptake of capecitabine loaded DS-NPs by U87MG glioblastoma cells were proved by Prussian blue staining and TEM, qualitatively. DS-NPs are suitable candidates for delivery of the hydrophilic drugs in cancer treatment and due to positive charge of the dextran-spermine, the uptake of the hydrophilic drugs by the cancerous cells was improved.

## Introduction

Many kinds of drugs developed for biologic and clinical applications have short half-life period because of their *in-vivo* degradation. On the other hand, some of drugs show contrary side effects in high-dose administration. Thus, developing know-how to evade or reduce these problems is important. Polymeric materials for drug delivery have been widely applied in research trials (1-3), including altered surface of drugs by a hydrophilic biopolymeric layer to protect drug against elimination from blood circulation (4-6) and these materials engineering progresses have been significantly prosperous in decreasing the essential dose and frequency of drugs for therapeutic efficiency with less adverse effects (7).

**Figure 1:**
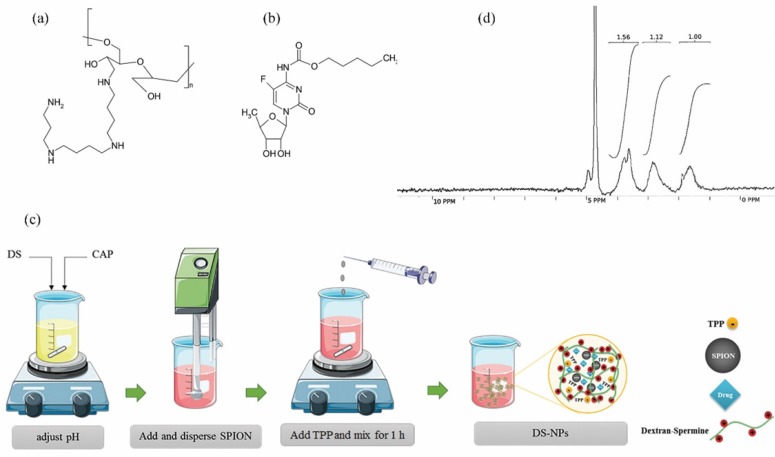
Chemical structure of dextran-spermine (a) and capecitabine (b); schematic diagram for preparation of capecitabine loaded magnetic dextran-spermine nanoparticles (DS-NPs) (c) and H-NMR spectra of synthesized dextran-spermine (d

Dextran as a biopolymer with low immunogenicity has been widely investigated for potential applications in DNA/drug delivery to cancerous cells (7-9). Cationic dextran was synthesized by spermine conjugation to the hydroxyl groups of dextran (10). Dextran-spermine is an appropriate carbohydrate polymer for gene delivery because of its positive surface charge (Figure 1a).

In recent years, super paramagnetic iron oxide nanoparticles (SPION) have revealed great possible uses in many biological arenas and there are so many reports on their application in drug delivery systems (11), including targeted drug delivery (12, 13), tissue healing, hyperthermia for cancer treatment (14, 15) and magnetic resonance imaging (MRI) (16, 17). Biocompatible polymer coating of SPION represent an extended blood circulation time with increased efficacy for diagnosis of the cancerous cells. Additionally, targeting of the nanoparticles to the specific organism or particular cell is possible by efficient conjugation of bioactive moieties, for instance enzymes, antibodies, peptides, and different antagonists, to these nanoparticles (18).

Polymeric nanoparticles can be prepared by different methods. One of the approved, simple, mild and less toxic methods to formulate nanoparticles from cationic polymers is the ionic gelation with tri-polyphosphate (TPP) (1, 19). 

Capecitabine (Figure 1b) is a hydrophilic pro-drug that can be converted to 5-fluorouracil (5-FU) in body tissues by enzymatic processes. It is broadly administered for patients who are involved in brain metastatic colorectal cancer and breast cancer. Preparation of a sustained release dosage form of capecitabine is necessary, due to high suggested regular dose (150 mg/m^2^), short elimination half-life (30-60 min) (20), and undesirable properties such as bone-marrow depression, cardio toxicity, diarrhea, nausea and vomiting, stomatitis, dermatitis, *etc.* related to this anticancer drug (21).

Because of the effective cellular internalization and recognition of cationic magnetic dextran-spermine nanoparticles by MRI (22), it was assumed that capecitabine (as an antineoplastic drug) loaded dextran-spermine nanoparticles can be considered as an appropriate system for drug delivery to cancerous cells. It is postulated that these nanoparticles will be more cytotoxic than free capecitabine by transporting the nanoparticles to U87MG glioblastoma cells with less side effects.

Hence, the aim of this study was encapsulation of capecitabine and SPIONs in dextran-spermine nanoparticles (DS-NPs) by ionic gelation to formulate a drug delivery system with enhanced efficacy for recognition of the cancerous cells. The effects of several factors comprising: (1) Dextran-spermine (DS) concentration, (2) TPP concentration, (3) TPP addition rate, (4) pH of DS solution and (5) SPIONs/DS mass ratio on the capecitabine loaded magnetic nanoparticles properties were investigated to maximize drug entrapment efficiency and zeta potential and minimize average size of nanoparticles. The fractional factorial design was applied to obtain dextran-spermine nanoparticles with desirable properties, followed by testing them against cancerous cells.

## Experimental

*Materials*

Paraformaldehyde, potassium ferrocyanide, hydrochloric acid, and dimethyl thiazol diphenyl tetrazolium bromide (MTT) were purchased from Merck (Germany). Dextran (MW = 40 kDa), sodium tri-polyphosphate, potassium periodate, Tri-nitrobenzene sulfonic acid (TNBS) solution, spermine and sodium bromide were obtained from Sigma-Aldrich (Germany). SPIONs (Fe_3_O_4_ aq, 8 nm) was supplied by Plasma Chem (Berlin, Germany). 

Human glioblastoma cells (U87MG) were obtained from Pasteur Institute (Tehran, Iran), and cultivated in a-MEM (alpha-modified minimum needed medium, Invitrogen, Carlsbad, CA, USA) comprising 10% fetal bovine serum (FBS). 

Capecitabine as an anticancer drug that was kindly gifted by Toseeh Danesh Company (Tehran, Iran). All other chemicals and solvents were common analytical-grade reagents. 

*Methods*

*Synthesis of dextran-spermine conjugate*

Dextran–spermine conjugate was synthesized as described by Azzam *et al.* (10). To obtain oxidized dextran, dextran was dissolved in doubly distilled water (DDW) and potassium periodate was added to dextran solution at 1:1 mol ratio (IO_4_^-^/dextran), and mixed vigorously at room temperature in the dark place for 6-8 h to obtain a clear-yellow solution. To obtain a purified oxidized dextran in the form of white powder, the iodate (IO_3_^-^) and unreacted periodate (IO_4_^-^) were separated by dialyzing the resulting poly aldehyde derivatives against DDW for 48 h at 4 ºC using cellulose tube (MWCO = 12000, Sigma) followed by freeze-drying. To conjugate spermine to dextran, oxidized dextran solution in DDW was slowly added drop wise to the spermine basic solution, prepared in 0.1 M borate buffer (pH 11), for a period of 5-7 h and mixed gently at room temperature for 24 h. Amine-based conjugates (reduced), were obtained by addition of NaBH_4_ to the mixture in two step (mixing 48 h for first step and 24 h for second step) at room temperature. The resulting mixture was dialyzed against DDW at 4 ºC using cellulose dialyze tubing bag (MWCO = 3500, Membrane Filtration Products Inc., San Antonio, TX, USA) and freeze-dried to get a yellow powder of dextran-spermine. The primary amine content of synthesized dextran-spermine was determined by TNBS method, H-NMR and elemental analysis.

*Experimental design studies*

Based on the literature report on preparation of cationic nanoparticles (22-24), five factors at two levels were selected for design of experiments (Table 1). Then five factor 2-level 2 ^(5-1)^ fractional factorial design was applied to set 19 experimental conditions given in Table 2. Each response was analyzed by analysis of variance (ANOVA) using Design-Expert software^® ^(Version 8.0.1, Stat Ease Inc.). The significant effects of each factor were determined through a regression model given by Equation (1):

Y_n _= a_0 _+ a_1_A + a_2_B + a_3_C + … + a_12_AB + a_13_AC + …                     (1)

Where Y_n_ represents the dependent variables of particle size (PS), entrapment efficiency (EE) and zeta potential (ZP), while A, B, C… are the coded factors and factors combination such as AB indicates the two way interaction between two factors in that term. a_0_ is the mean of responses while a_n_ is the coefficient related to the n^th^ factor. 

Using such experimental design, the dependence of each response on dependent variables and their interactions in the resolution of Vis investigated (25).

*Preparation of capecitabine loaded DS-NPs*

The capecitabine loaded DS-NPs were prepared by ionic gelation at different experimental conditions given in Table 2. As shown in Figure 1c, dextran-spermine and capecitabine were separately dissolved in DDW and then mixed. Then, the pH of this solution was adjusted by addition of 1N HCl to activate the amine group of the dextran-spermine. Then, SPIONs were dispersed in the resultant solution by sonication for 5 min followed by addition of 0.4 mL TPP solution as a cross-linker (TPP solution pH is about 8) at a pre-determined rate, given in Table 2, to the resulting suspension under magnetic stirring. The obtained suspension was further stirred for 1 h to assure nanoparticles formation.

*Nanoparticles characterization*

*Fourier transforms infrared (FT-IR) spectroscopy*

The chemical interactions between capecitabine and DS and entrapped SPIONs were studied using FT-IR spectroscopy. So, powders of capecitabine loaded magnetic dextran-spermine nanoparticles, DS and SPIONs were individually pressed to form corresponding tablets. The spectra was discovered by mixing with KBr over a span of 3900-400 cm^-1^ and documented by FT-IR spectrophotometer (Perkin Elmer Spectrometer Frontier, America).

*Particle size and zeta potential measurement of DS-NPs*

The particle size and surface charge of DS-NPs were evaluated on the basis of dynamic light scattering (DLS) procedure using a Zetasizer (Nano-ZS; Malvern Instruments, Malvern, UK). To prepare a uniform suspension of nanoparticles for DLS measurement, the DS-NPs were dispersed in 1 mL DDW by sonication for 1 min.

*Morphological studies of DS-NPs*

The morphology of DS-NPs was studied by scanning electron microscope (SEM) (EM3200, 26 kV, KYKY, China) and transmission electron microscope (TEM) (CM30, 300 kV, Philips). 

For this purpose, 100 μL of dispersed DS-NPs in 2 mL DDW was sonicated for 1 min and a drop of resulting suspension was placed on the SEM stub and left in air to dry at room temperature. Afterward, dried DS-NPs were coated with gold and their morphology was determined by SEM. Before the assessment of DS-NPs by TEM, the DS-NPs suspension was dropped on a copper grid which covered with a formal carbon membrane. 

*Vibration sample magnetometer (VSM)*

VSM-MDK6 was applied to determine the magnetic properties of DS-NPs and SPIONs as control sample. The field dependence and saturated DS-NPs magnetization were recorded under circulate magnetic field in the ranged of -1.5 - 1.5T at 300 K.

*Entrapment efficiency measurement*

To measure the entrapment efficiency of drug, dispersion of DS-NPs was centrifuged at 30000×g and 14 °C. Then, the supernatant was separated and analyzed by HPLC method to determine free drug concentration (26). The entrapment efficiency of capecitabine (EE) was computed by Equation (2):

$\mathrm{EE}=\frac{W_{1}-W_{2}}{W_{1}}\times 100\%$

(2)

Where W_1_ is the initial mass of drug used for nanoparticles preparation and W_2 _is mass of free drug in the supernatant after centrifugation.

*HPLC analysis of capecitabine*

The concentration of capecitabine was quantified by reverse phase high performance liquid chromatography (MERCK HITACHI). Chromatographic separation was performed on C18 Perkin Elmer column (250 mm × 4.6 mm, 5 μm) at an isocratic mode and flow rate of 1 mL/min with UV detection at 230 nm. The mobile phase consisted of methanol: acetonitrile: water at a specific ratio of 80:18:2 v/v (26). 

*Optimization of DS-NPs preparation*

The optimum values of the factors that affect DS-NPs preparation were determined by the simultaneous optimization method (27). A singular desirability function d_i _was considered for each response that can be altered in the range 0 < d_i _< 1. Then, the design factors were carefully selected to yield the best desirability out of Equation (3):

(3) $$D={\left(d_{1}d_{2}d_{3}\ldots d_{m}\right)}^{1/m}$$

Where m is the number of responses, D and d_i _are the total and singular desirability, respectively.The aim of optimization was to enhance entrapment efficiency of hydrophilic drug, capecitabine in DS-NPs, and surface charge of DS-NPs as well as particle size minimization. So, DS and TPP concentrations, TPP addition rate, pH of DS solution and SPIONs/DS mass ratio were restricted to the surveyed scope of the experimentations in the model to attain maximum desirability.

*In-vitro release of capecitabine*

The optimum formulation was selected to study capecitabine release in acetate buffer (AB) at pH 4.8 and phosphate buffer saline (PBS) at pH 7.4. The acidic medium (AB at pH 4.8) was selected to simulate the microenvironment of endosomal and lysosomal compartments of cancerous cells. Hence, 5 mg of capecitabine loaded DS-NPs were suspended in both media and shaken in an orbital shaker bath at 37 ºC and 80 rpm. 

The supernatant was centrifuged at 17000 rpm for 10 min, at predetermined time intervals to separate capecitabine released from DS-NPs. To keep sink condition, drug loaded NPs were dispersed insufficient amount of fresh media at each time interval. Moreover, the amount of capecitabine in supernatant was quantified by HPLC technique based on the calibration curve (26). The *in-vitro* release assessment was carried out in triplicate and corresponding data are reported as mean ± SD.

*Cell line studies*

Glioblastoma U87MG cell lines were obtained from Pasteur Institute (Tehran, Iran). U87MG cells were cultured in Dulbecco’s modified Eagle medium (DMEM) and FBS (10%) and incubated in moist environment of 5% CO_2 _at 37 ºC. This medium was substituted every other day and the cells were collected by tripsynization via 0.25% Trypsin-EDTA solution.

*Cytotoxicity studies*

Cell viability in the presence of DS-NPs was tested by MTT assay. For this purpose, 5000 U87MG cells were placed in the 96-well plates and incubated for 12 h to permit cell attachment. Then, the wells medium was substituted with fresh medium consisted of various concentrations of capecitabine, capecitabine loaded DS-NPs, and blank DS-NPs and incubated for another 24 and 48 h. Then, 10 µL of MTT solution (5 mg/mL) was added to each well and incubated for 4 h at 37 ºC. After that, the medium was removed and formazan crystals were dissolved in 150 µL DMSO for 10 min while shaking. The absorbance of each well medium was investigated by a micro plate reader at a wavelength of 540 nm. The cell viability (%) for each sample was compared with the control well (U87MG cells without DS-NPs).

*Cellular uptake of DS-NPs*

Prussian blue staining experiment and TEM imaging were performed to study the intracellular uptake of capecitabine loaded magnetic dextran-spermine nanoparticles. For this purpose, 8000 U87MG cells were placed into each well of 96-well plates and incubated for 12 h to cells attachment. Then, the culture medium was replaced with 1 mL of fresh medium comprising 0.1 mg/mL DS-NPs and incubated for 14 h. Then, to remove any free DS-NPs, the cells were rinsed with PBS buffer and stabilized with 4% paraformaldehyde for 40 min. Consequently, washing with PBS, the cells were incubated in 2 mL Prussian blue solution at 37 ºC for 30 min. Prussian blue solution was prepared by mixing of equal volume of hydrochloric acid solution (2%) and potassium ferrocyanide (II) trihydrate (2%). Finally, stained cells were rinsed thrice and imaged by a light microscope (magnification of 200).To study the internalization of DS-NPs into the cells by TEM imaging, the U87MG cells were incubated with 0.1 mg/mL DS-NPs for 24 h and rinsed with PBS. The cells were prepared for TEM imaging by standard protocol and then observed with a TEM (CM30, 300 kV, Philips) at 80 kV.

## Results and Discussion

*Characterization of dextran-spermine*

Reductive-amination technique was performed to conjugate spermine to oxidized dextran and resulting polycation was assessed via TNBS, H-NMR and elemental analysis methods. The primary amine content of dextran-spermine conjugate in borate buffer was 0.83 mmol/g which is comparable with those reported in literature (28, 29).

Elemental analysis was carried out for qualitative and quantitative determination of the amine group coupling to oxidized dextran. Only carbon (40.73%) and hydrogen (5.67%) atoms exist in pure oxidized dextran structure. So, the nitrogen is integrated in the dextran structure by conjugating the spermine to the oxidized dextran. Nitrogen proportion for DS was estimated to be 7.38% compare to 10.84% reported in literature (29). The H-NMR spectra were as follow: 1.63 (m, 4H, dextran-CH_2_NH (CH_2_)_3_NHCH_2_CH_2_CH_2_CH_2_NH(CH_2_)_3_NH_2_), 1.77 (m, 4H, dextran-CH_2_NHCH_2_CH_2_CH_2_NH (CH_2_)_4_NHCH_2_CH_2_CH_2_NH_2_), 2.79 (m,12H, dextran-CH_2_NHCH_2_CH_2_CH_2_NHCH_2_ (CH_2_)_2_CH_2_NHCH_2_CH_2_CH_2_NH_2_), 3.10-4.22 (m, sugar hydrogens) and 4.71 (m, 1H, anomeric hydrogens of sugars) and the H-NMR image is shown in Figure 1d.

**Figure 2:**
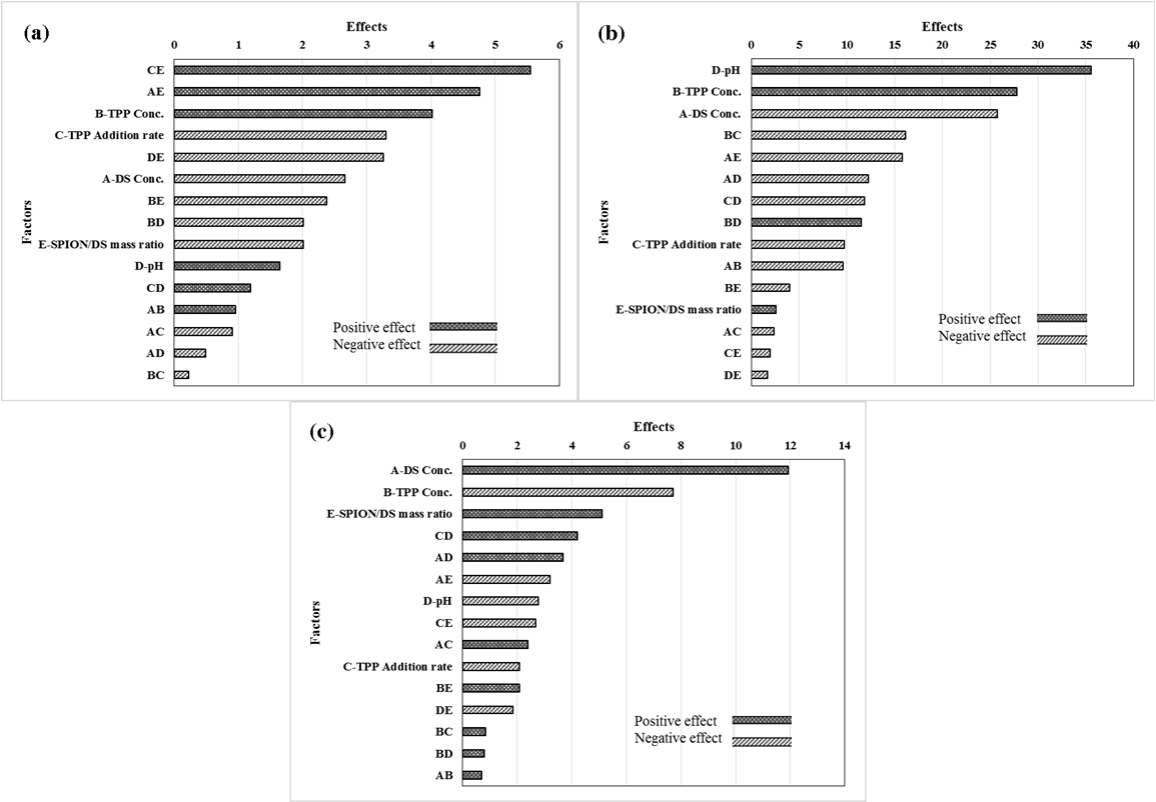
Pareto chart of the effective factors on the entrapment efficiency of capecitabine in cationic polymeric nanoparticles (a); Pareto chart of the factors affecting in particle size of capecitabine loaded cationic polymeric nanoparticles (b); Pareto chart of the factors affecting in zeta potential of capecitabine loaded cationic polymeric nanoparticles (c

**Figure 3:**
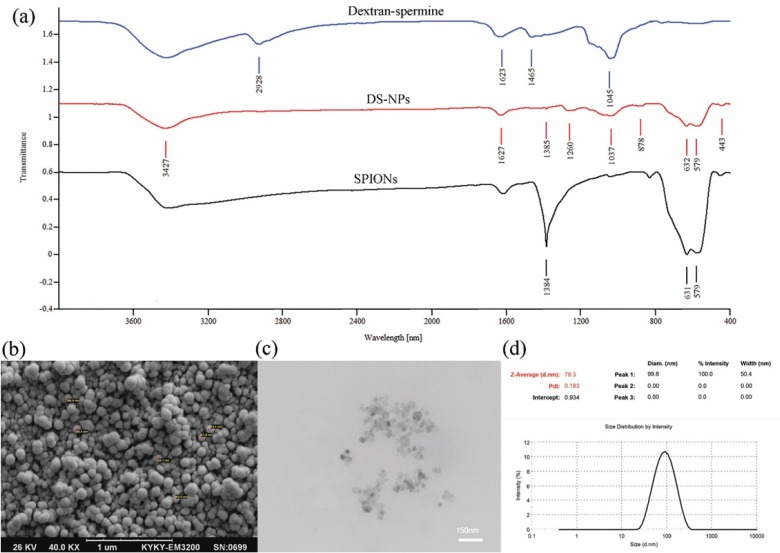
FT-IR spectra of dextran-spermine, DS-NPs and SPIONs (a); SEM (b) and TEM (c) images of capecitabine loaded DS-NPs; Size and the polydispersity of DS-NPs measured by DLS (d

**Figure 4.**
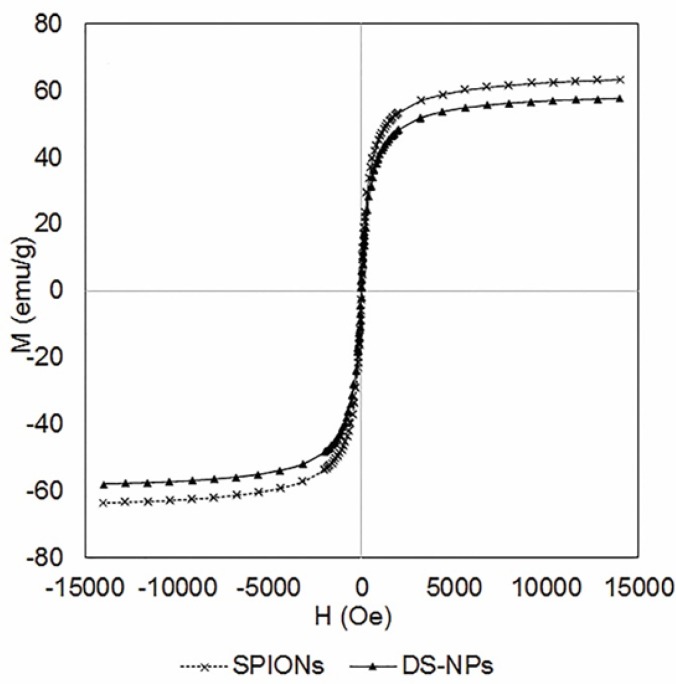
The magnetic behavior (VSM) of SPIONs and DS-NPs (300 K

**Figure 5:**
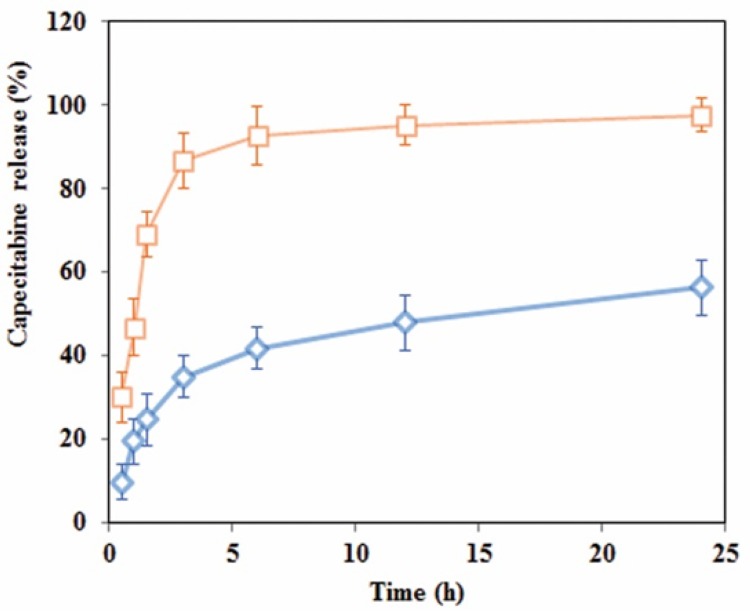
Release profile of capecitabine from cationic dextran-spermine nanoparticles in two: (□) acidic (pH 4.8) and (◊) neutral (pH 7.4) media

**Figure 6:**
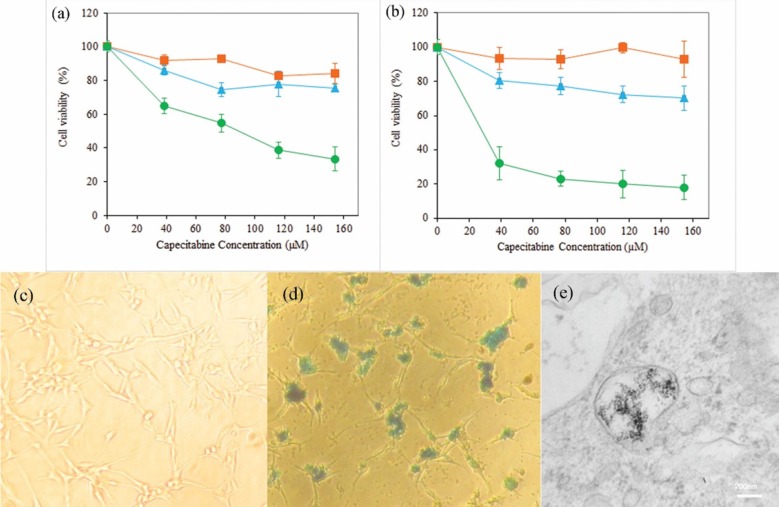
Viability of U87MG cells determined by MTT assay after incubation with neat DS-NPs (▪), free capecitabine (▲) and capecitabine loaded DS-NPs (●) at different concentrations of capecitabine (0-160 µM) for 24 h (a) and 48 h (b) (n = 3); Prussian blue staining images of U87MG cells after 14 h incubation with control group (c) and capecitabine loaded DS-NPs (d) at concentration of 0.1 µg/mL; TEM image of U87MG cells fixed after incubation with DS-NPs (e

**Table 1:** Levels of the factors used in experimental design for preparation of capecitabine loaded DS-NPs according to the half fractional factorial 2 ^(5-1)^ design.

**Factors**	**Units**	**Levels**	
		**Low (-1)**	**High (+1)**
A- Dextran-spermine Conc. (DS)	mg/mL	2.00	4.00
B- TPP Conc. (TPP)	mg/mL	0.50	1.00
C- TPP Addition rate (Rate)	mL/min	35.00	60.00
D- pH (pH)	-	3.00	5.00
E- SPION/DS mass ratio (SPION/DS)	-	0.50	0.75

* A- Dextran-spermine concentration (DS); B- Tri-polyphosphate concentration (TPP); C- Tri-polyphosphate addition rate (Rate); D- pH of dextran-spermine solution (pH); E- Super paramagnetic iron oxide nanoparticle/dextran-spermine mass ratio (SPION/DS).

**Table 2:** Responses values for particle size (nm), zeta potential (mV) and entrapment efficiency (%) of DS-NPs according to the half fractional factorial 2 ^(5-1)^ design

**Std order**	**Factors**						**Responses**		
		**DS(mg/mL)**	**TPP(mg/mL)**	**rate(mL/min)**	**pH** **-**	**SPION/DS** **-**	**PS (nm)**	**ZP(mV)**	**EE (%)**
S_1_	- - - - +	2.00	0.50	35.00	3.00	0.75	76	+37.90	13.23
S_2_	+ - - - -	4.00	0.50	35.00	3.00	0.50	64	+35.50	12.92
S_3_	- + - - -	2.00	1.00	35.00	3.00	0.50	96	+15.00	27.13
S_4_	+ + - - +	4.00	1.00	35.00	3.00	0.75	77	+33.30	20.15
S_5_	- - + - -	2.00	0.50	60.00	3.00	0.50	72	+20.30	10.55
S_6_	+ - + - +	4.00	0.50	60.00	3.00	0.75	72	+32.40	15.70
S_7_	- + + - +	2.00	1.00	60.00	3.00	0.75	100	+19.40	17.89
S_8_	+ + + - -	4.00	1.00	60.00	3.00	0.50	76	+24.40	11.35
S_9_	- - - + -	2.00	0.50	35.00	5.00	0.50	99	+17.50	26.12
S_10_	+ - - + +	4.00	0.50	35.00	5.00	0.75	80	+33.90	14.49
S_11_	- + - + +	2.00	1.00	35.00	5.00	0.75	183	+18.20	11.37
S_12_	+ + - + -	4.00	1.00	35.00	5.00	0.50	139	+24.10	23.28
S_13_	- - + + +	2.00	0.50	60.00	5.00	0.75	117	+20.80	17.04
S_14_	+ - + + -	4.00	0.50	60.00	5.00	0.50	83	+39.60	9.38
S_15_	- + + + -	2.00	1.00	60.00	5.00	0.50	135	+10.20	22.80
S_16_	+ + + + +	4.00	1.00	60.00	5.00	0.75	80	+31.60	17.59
S_17_	0 0 0 0 0	3.00	0.75	47.50	4.00	0.63	90	+32.80	11.19
S_18_	0 0 0 0 0	3.00	0.75	47.50	4.00	0.63	89	+29.67	17.98
S_19_	0 0 0 0 0	3.00	0.75	47.50	4.00	0.63	96	+25.78	16.97

* A- Dextran-spermine concentration (DS); B- tripolyphosphate concentration (TPP); C- tripolyphosphate addition rate (Rate); D- pH of dextran-spermine solution (pH); E- Super paramagnetic iron oxide nanoparticle/dextran-spermine mass ratio (SPION/DS); Particle size (PS); Zeta potential (ZP); Entrapment efficiency (EE).

**Table 3:** Characteristics of fitted model to the responses

**Dependent Factors**	***P*** **-value**	**R** ^2^	**Adj. R** ^2^	**Lack of Fit**
EE (%)	0.0059	0.91	0.79	Insignificant (*P*-value > 0.1)
PS (nm)	0.0002	0.99	0.96	Insignificant (*P*-value > 0.1)
ZP (mV)	0.0019	0.94	0.84	Insignificant (*P*-value > 0.1)

* EE: Entrapment efficiency; PS: Particle size; ZP: Zeta potential.

**Table 4:** Comparison between predicted and experimental values for the optimal formulation of capecitabine loaded DS-NPs

	**EE (%)**	**PS (nm)**	**ZP (mV)**
Predicted	23.43	73	+22.5
Experimental	26.09	79	+22.8
Absolute relative deviation	11.35%	8.22%	1.33%

* EE: Entrapment Efficiency; PS: Particle Size; ZP: Zeta Potential.

*Effect of process factors on the entrapment efficiency of capecitabine loaded DS-NPs*

Based on fractional factorial design, 19 experiments were conducted as shown in Table 2 and for each response, the analysis of variance (ANOVA) was implemented with significant probability of 0.05. The goodness of fit to experimental data with statistical model was tested by the high coefficient of determination, R^2^ (Table 3), and the randomness of the residuals. 

The entrapment efficiency of the capecitabine in DS-NPs as a function of the effective factors is given by Equation (4) as follow:

Entrapment Efficiency (%) = 48.4 - 13.2 × DS + 47.9 × TPP - 1.2 × Rate + 12.0 × pH - 68.6 × SPION/DS + 19.0 × DS × SPION/DS - 4.0 × TPP × pH - 38.1 × TPP × SPION/DS + 1.8 × Rate × SPION/DS - 13.1 × pH × SPION/DS                    (4)

As presented in Figure 2a, the positive effects mean that when the amount of factor increases in the studied range, the response increased and this increment is favorable for the response, while negative effects indicates that the response will be decreased by decreasing the amount of factor in the studied range, and this decrement is desirable. The degrees of the effects specify the relative influences of the factors in the response. The *P*-value of each term of the factors, denotes the probability of error which is required in accepting the practical outcomes. In consequence, the smaller *P*-value, the corresponding coefficient term is the more significant. The effects of factors, containing main and binary interactions of the factors, on entrapment efficiency of capecitabine in the magnetic dextran-spermine nanoparticles, are illustrated in Figure 2a.

TPP concentration and pH of the dextran-spermine solution have positive effect on the entrapment efficiency in the considered range. So, high entrapment efficiency of capecitabine will be achieved at higher levels of TPP concentration and pH of DS solution. The decrease in the entrapment efficiency with the increase in DS concentration from 2.0 to 4.0 mg/mL, can be related to the increases in solution viscosity with increasing DS concentration. It has been reported that drug molecule movement within polymer molecular chains will be obstructed due to high viscosity related with increased polymer concentration, and prevented entrapment of drug (30, 31).

As illustrated in Figure 2a, capecitabine entrapment efficiency improved through increasing TPP concentration. One clarification is that high TPP concentration (pH 8.9) at constant DS concentration, may cause a growth in pH value of the mixture, with a subsequent rise of totally negative charge transport by the capecitabine molecules (with an isoelectric point of 2.43), which in turn augments the electrostatic interactions between DS and capecitabine molecules (23). It is noticeable that the entrapment efficiency increased by increasing the pH of DS, as shown in Figure 2a. It can be related to more negative charge at pH values greater than capecitabine isoelectric point and consequently, increased ionic interaction of DS with TPP and capecitabine (24).

*Effect of process factors on particle size of capecitabine loaded DS-NPs*

The effects of main and binary interactions of factors on the particle size are shown in Figure 2b. The particle size decreased by increasing DS concentration and the rate of TPP addition due to more available sites for particles nucleation and accelerated cross linking of activated amine groups in DS by phosphate ions for particle formation.

TPP concentration is another factor that affects the formation and characteristics of DS-NPs throughout ionic gelation process. It has been reported that the particle size of chitosan nanoparticles decreased by decreasing TPP concentration (32). 

We observed that the turbidity of the nanoparticles suspension increased by increasing of TPP concentration. Aggregates of chitosan nanoparticles with large size were formed when TPP concentration was high (30). From theoretical point of view, fast gelation of the cationic polysaccharide polymers upon contact with TPP as a cross linker, is due to inter- and intra-molecular linkage between amino groups of DS and phosphate groups of TPP. At low concentration of TPP, it may be consumed mainly by intra-molecular cross-linking with DS molecules to form smaller nanoparticles. But at high concentration of TPP, the accessible molecules of TPP increases with consequent intermolecular interactions of them with DS molecules of adjacent nanoparticles that lead to formation of larger nanoparticles. 

The formation of DS-NPs by ionic gelation was pH dependent and nanoparticles with smaller size and higher zeta potential were formed at low pH values. The particle size of nanoparticles was increased by increasing the pH value from 4.0 to 6.0. 

The same trend for chitosan nanoparticles formation was also described by Shu and Zhu (33). At lower pH values, a higher zeta potential and stronger linkage between TPP and NH_3_^+^ is due to higher protonation of –NH_2_ moiety that resulted compact nanoparticles with small particle size. In addition, the strong repulsion between nanoparticles with higher zeta potential prevented their aggregation. At higher pH values, larger nanoparticles would be formed due to the relatively weak interactions between DS and TPP molecules (34). 

The dependence of DS-NPs size on effective factors can be described by the following statistical Equation (*P*-value < 0.05):

Particle size (nm) = -327.6 + 70.0 × DS + 143.8 × TPP + 3.7 × Rate + 41.4 × pH + 199.8 × SPION/DS - 19.2 × DS × TPP - 0.1 × DS × Rate - 6.1 × DS × pH - 63.2 × DS × SPION/DS - 2.6 × TPP × Rate + 22.9 × TPP × pH - 0.5 × Rate × pH                    (5)

*Effect of process factors on zeta potential of capecitabine loaded DS-NPs*

Figure 2c shows the pareto chart of factors that are effective on the zeta potential of DS-NPs. In the studied range, the zeta potential of capecitabine loaded DS-NPs improved with DS concentration. As described earlier, the interaction between protonated amine group of DS (-NH_3_^+^) with poly anionic phosphate groups of TPP is responsible for formation of DS-NPs by ionic gelation. The zeta potential of DS-NPs is regulated by the poly anionic phosphate groups of TPP via the degree of neutralization of -NH_3_^+^. As a result, in the greater DS concentration, there are more available -NH_3_^+^ in dextran-spermine solution to be neutralized and leads to higher zeta potential of DS-NPs. Hu *et al.*, have reported the linear relation between the chitosan concentration and zeta potential of the nanoparticles (35).

The zeta potential of DS-NPs decreased by increasing the TPP concentration from 0.5 to 1.0 mg/mL due to neutralization of cations on the surface of particles by poly anionic phosphate groups of TPP throughout the linking or accumulation of the nanoparticles.

One of the factors that had positive effect on zeta potential is the SPION/DS mass ratio. The SPION used in preparation of the nanoparticles, had positive surface charge and it is reasonable to assume the increase of zeta potential of DS-NPs by increase of SPION content in the reaction mixture with possible presence on the surface of polymeric nanoparticles. The statistical relation (*P*-value < 0.05) to describe the dependence of zeta potential on the effective factors is given by:

Zeta Potential (mV) = 50.6 + 2.1 × DS - 36.3 × TPP - 0.5 × Rate - 14.9 × pH + 74.8 × SPION/DS + 0.1 × DS × Rate + 1.8 × DS × pH - 12.9 × DS × SPION/DS + 33.4 × TPP × SPION/DS + 0.2 × Rate × pH - 0.9 × Rate × SPION/DS - 7.4 × pH × SPION/DS                    (6)

*Optimum formulation for preparation of drug loaded DS-NPs*

To find the optimum condition for preparation of capecitabine loaded dextran-spermine, the optimization was based on the criterion of desirability functions for the three responses: Entrapment efficiency of capecitabine and zeta potential and particle size of DS-NPs. The best formulation in the studied range, was found to be at 2 mg/mL DS concentration, 0.75 mg/mL TPP concentration at an addition rate of 35 mL/min, pH 3 of polymer solution and SPION/DS mass ratio of 0.5. The capecitabine loaded DS-NPs were prepared at optimum condition and the corresponding experimental results were fitted to the fractional factorial model. The investigational outcomes in Table 4 indicates that model computations agreed with the experimental results.

*FT-IR spectroscopy*

To evaluate the interactions among capecitabine, SPIONs and dextran-spermine, the FT-IR spectra of SPIONs, capecitabine loaded magnetic dextran-spermine nanoparticles and dextran-spermine polymer are displayed in Figure 3a. The FT-IR spectrum of SPIONs displays the peak situated at 1384 cm^-1^ which can be allocated to C-H bending vibration mode and the representative peak at 579-630 cm^-1 ^corresponds to the stretching vibration mode of Fe_2_O_3 _(36). The bending mode of H-O-H adsorbed at the SPIONs surface can be directed as a weak band around 1615 cm^-1^. The strength of absorption peaks of 580 and 631 cm^-1^ for SPIONs were significantly reduced by entrapment in dextran-spermine. Moreover, there was no peak in dextran-spermine spectrum in the range of 650-400 cm^-1 ^due to the entrapment of SPIONs in dextran-spermine matrix, as an indication of effective loading of SPIONs in the dextran-spermine polymeric network.

*Shape and morphology of nanoparticles*

The size and surface morphology of DS-NPs were determined by dynamic light scattering (DLS), SEM, and TEM images. SEM (Figure 3b) and TEM (Figure 3c) images show that DS-NPs with a size of 79 nm, were spherical and evenly distributed. Additionally, the DS-NPs size determined by TEM and SEM agreed well with DLS results, as shown in Figure 3d. Also, the size of neat DS-NPs (68 nm) is smaller than capecitabine loaded DS-NPs (79 nm) and it is agreed well with result reported by Mohammad-Taheri *et al.* (22).

*Magnetization property of DS-NPs*

The magnetic properties of DS-NPs were assessed by VSM under a magnetic field to approve their potential applications as theranostic system for MR imaging and drug delivery. Figure 4 indicates that DS-NPs have super paramagnetism property without magnetic hysteresis at 300 K and its saturation magnetization value was 57.8 emu/g at 1.5T, which is lower than that of SPIONs (63.3 emu/g). It can be due to the thick polymeric shells surrounding the SPIONs. Shen *et al.* have reported similar result by functionalizing RGD to polymeric magnetic nanoparticles (12). However, DS-NPs have considerably higher saturation magnetization values as compared to those reported by other researchers, *e.g.* 29.1 emu/g (37), 27 emu/g (12) and 14 emu/g (38) that means they would be more appropriate for clinical applications.

*In-vitro release of capecitabine*

Drug release experimentations were performed in the buffer medium with neutral and acidic pH to mimic drug release profile in cancerous cell microenvironment via an HPLC technique. The calibration curve was constructed in a range of 0.06-1.2 mg/mL (R^2^ = 0.99) and accuracy and precision data were determined accordingly. The capecitabine release profile at pHs 4.8 and 7.4 are shown in Figure 5. As shown in this Figure, 98% of entrapped capecitabine in DS-NPs released within 3 h in acetate buffer at pH 4.8, while entrapped capecitabine release in neutral media within 24 h was 56% without burst effect.

Faster drug release at acidic medium, can be attributed to expanded structure of swollen network made of cationic DS polymer (39). The release kinetic of capecitabine from DS-NPs was analyzed by an empirical Equation, known as Korsmeyer-Peppas relation (Equation (7)) (40) as follow:

$\frac{M_{t}}{M_{\infty }}={\mathrm{kt}}^{n}$

(7)

Where M_t_/M_∞ _is the fractional drug release at time t, k is a constant related to diffusion coefficient of drug and geometry of the release device. The exponent of n is a factor that describes the release mechanism. n value of n = 0.5 stands for Fickian diffusion and 0.5 < n < 1.0 is an indicative anomalous diffusion due to polymer chain relaxation and n = 1 describes case II transport due to constant rate of swelling or erosion front (41). However, the mechanism of drug release is more sophisticated to be described with such a simple empirical relation. In this study, the value of n = 0.42 at neutral medium (R^2^ = 0.90) shows that the drug release controlled by Fickian diffusion mechanism, and at acidic medium, a value of n = 0.61 

(R^2^ = 0.96) indicates that a combination of diffusion, swelling and polymer chain cleavage degradation may be responsible for drug release from DS-NPs. Acidic environment or pH of living cells and tissues is one of the main factors that affects cell cycle progression, cell proliferation, and differentiation (42). It has long been proved that as compared with normal tissues, tumors of both animal and human have acidic microenvironment, due to elevated anaerobic along with aerobic glycolysis in tumors (43). As shown in Figure 5, drug release from DS-NPs at neutral environment had a sustained release behavior, while at acidic environment, a burst release occurred. Thus, it can be concluded that this kind of nanocarriers are promising candidates for antineoplastic drugs delivery to tumor sites. It is expected that the drug will be released slowly till the nanocarriers reach to the targeted site (tumor site) where the drug will be released upon its exposure to the acidic environment of tumor. 

*In-vitro cell investigation*

The cytotoxicity of neat and capecitabine-loaded DS-NPs and free drug were evaluated by MTT test on the U87MG glioblastoma cell line. Figures 6a and 6b display that neat nanoparticles and free drug did not have significant toxicity on these cells, and the capecitabine loaded DS-NPs were more toxic. Capecitabine is a hydrophilic anticancer drug that penetrates into cells by membrane transporters (44) and more capecitabine is required to have toxicity effect on these cells due to low potency of capecitabine. Nanoparticles can be taken up by cells via a clathrin mediated endocytosis route (45, 46). Hence, nanoparticles play a facilitator role for drug delivery into the cells. In addition, tumor cells have strong negative charge on their surface because of the presence of sialic acid, human chorionic gonadotropin and RNA residues with negative charge on them (47, 48). Accordingly, cationic nanoparticles interact electro statically with membrane of the cancerous cells and can be internalized more effectively into U87MG cell. Capecitabine release from positively charged DS-NPs that are attached or penetrated into the cells, provides more drug for membrane passage followed by accumulation in the interior of the cells. Both mechanism can be responsible for cytotoxicity of capecitabine loaded magnetic DS-NPs.

Effective uptake of anticancer drug carriers by cancer cells is important and the usefulness of magnetic NPs along with their biomedical fortune within cells extremely depend on their surface charges. For that reason, the cellular internalization of capecitabine and SPIONs loaded DS-NPs by human glioblastoma U87MG cells was investigated using Prussian blue staining to detect the distribution of intracellular iron. Figures 6c and 6d show the images of Prussian blue staining of DS-NPs that were freely taken up by U87MG cells as specified by blue points in the cytoplasm or round the nuclei. To extra confirmation of particles internalization, the TEM images of U87MG cells, incubated with DS-NPs, were taken. Figure 6e obviously indicates the uptake nanoparticles and presence of numerous vesicles comprising clusters of nanoparticles in the cytoplasm. 

The process of nanoparticles uptake, can be attributed to their adsorption to the surface of cancerous cells follow by generation of vesicles containing nanoparticles and consequent internalization of them by endocytosis (49).

## Conclusions

One of the appropriate methods in pharmaceutical technology researches to plan, select or to launch the smaller number of experimentations is the experimental design strategy to obtain the essential information by the most effective and accurate approach.

Fabrication of capecitabine loaded DS-NPs was carried out via ionic gelation method and statistical design of experiments were applied to determine the effects of process variables on the characteristics of magnetic polymeric dextran-spermine nanoparticles to obtain maximum drug entrapment efficiency and zeta potential as well as minimum particle size. 

The optimum condition for preparation of DS-NPs with a maximum capecitabine entrapment efficiency of 26.1% was found to be at DS concentration of 2 mg/mL, TPP concentration of 0.75 mg/mL with addition rate of 35 mL/min, pH 3 of dextran-spermine solution and SPION/DS mass ratio of 0.5. The release of capecitabine from dextran-spermine nanoparticles exhibited sustained release behavior up to 56% after 24 h at neutral pH, but a burst release up to 98% after 3 h in acidic pH was detected. This release behavior is desirable for delivery of an anticancer drug such as capecitabine to tumor cells. The *in-vitro *cytotoxicity and uptake of capecitabine and SPION loaded DS-NPs by glioblastoma U87MG cells as compared to free capecitabine, indicated that these nanocarriers are promising theranostic for MRI imaging and drug delivery for cancer treatment. Extra *in-vivo* studies are required to explore the potential application of these nanocarriers as a multi-functional theranostic system for identification and treatment of glioblastoma.
